# Effect of MgO Underlying Layer on the Growth of GaO*_x_* Tunnel Barrier in Epitaxial Fe/GaO*_x_*/(MgO)/Fe Magnetic Tunnel Junction Structure

**DOI:** 10.3390/s17102424

**Published:** 2017-10-23

**Authors:** Sai Krishna Narayananellore, Naoki Doko, Norihiro Matsuo, Hidekazu Saito, Shinji Yuasa

**Affiliations:** 1 National Institute of Advanced Industrial Science and Technology (AIST), Spintronics Research Center, Umezono 1-1-1, Central 2, Tsukuba, Ibaraki 305-8568, Japan; narayananellore-sk@aist.go.jp (S.K.N.); dokou.n@aist.go.jp (N.D.); matsuo-n@aist.go.jp (N.M.); yuasa-s@aist.go.jp (S.Y.); 2 Chiba Institute of Technology, 2-17-1 Tsudanuma, Narashino, Chiba 275-0016, Japan

**Keywords:** magnetic tunnel junction, epitaxial growth, gallium oxide, tunneling magneto-resistance, semiconductor

## Abstract

We investigated the effect of a thin MgO underlying layer (~3 monoatomic layers) on the growth of GaO*_x_* tunnel barrier in Fe/GaO*_x_*/(MgO)/Fe(001) magnetic tunnel junctions. To obtain a single-crystalline barrier, an in situ annealing was conducted with the temperature being raised up to 500 °C under an O_2_ atmosphere. This annealing was performed after the deposition of the GaO*_x_* on the Fe(001) bottom electrode with or without the MgO(001) underlying layer. Reflection high-energy electron diffraction patterns after the annealing indicated the formation of a single-crystalline layer regardless of with or without the MgO layer. Ex situ structural studies such as transmission electron microscopy revealed that the GaO*_x_* grown on the MgO underlying layer has a cubic MgAl_2_O_4_-type spinel structure with a (001) orientation. When without MgO layer, however, a Ga-Fe-O ternary compound having the same crystal structure and orientation as the crystalline GaO*_x_* was observed. The results indicate that the MgO underlying layer effectively prevents the Fe bottom electrode from oxidation during the annealing process. Tunneling magneto-resistance effect was observed only for the sample with the MgO underlying layer, suggesting that Ga-Fe-O layer is not an effective tunnel-barrier.

## 1. Introduction

Magnetic tunnel junctions (MTJs) have been intensively studied for various applications including magnetic sensors [1,2,3,4,5]. In MTJs, tunneling magnetoresistance (MR) ratio is one of the most important performance indexes and defined as (*R*_AP_ − *R*_P_)/*R*_P_ where *R*_P_ and *R*_AP_ are the resistances between the two ferromagnetic (FM) electrodes with parallel and antiparallel magnetization alignments, respectively. Fabrication of epitaxial structure is the key to achieving a high MR ratio because the coherent spin-polarized tunneling in fully epitaxial MTJs yields giant MR ratios [6,7,8], even at room temperature (RT), as reported in MTJs with insulating tunnel-barriers such as MgO [9,10,11], MgAl_2_O_4_ [12], and MgGa_2_O_4_ [13].

Semiconductors (SC) have great potential as the tunnel-barrier of MTJ for a low resistance-area product [14,15] because of its rather narrow band-gap, compared with insulators. Also, fully single-crystalline FM/SC/FM structure is one of the important building blocks of a vertical-type spin field-effect-transistor having nonvolatile memory functionality [16,17,18,19]. Here, the FM layer and SC layer each respectively act as source/drain electrodes and channel layer of the FET. Note that it is impossible to realize this device by using an insulator as the channel layer. In our previous studies, we have reported high MR ratios up to ~100% at RT in fully epitaxial Fe(001)/GaO*_x_*(001)/MgO(001)/Fe(001) MTJs where GaO*_x_* is a wide-gap semiconductor with a MgAl_2_O_4_-type cubic spinal structure (γ phase) [20,21]. Thanks to the coherent spin-polarized tunneling, the observed MR ratio is several times higher than those reported in MTJs consisting of polycrystalline FM electrodes with an amorphous GaO*_x_* barrier (at most ~22% at RT) [22,23,24]. This is the highest value among the reported MTJs with a SC barrier at RT [14,15,22,23,24,25,26,27,28,29,30,31]. It was found in the fully epitaxial MTJs that the growth of a few monoatomic (ML; 1 ML = 0.21 nm) MgO(001) underlying layers on the Fe(001) bottom electrode are indispensable to realize a high MR ratio and that a tunneling MR (TMR) effect cannot be observed without the MgO underlying layer. Cross-sectional observations of the Fe/GaO*_x_*/(MgO)/Fe showed sharp barrier/electrode interfaces without having the Fe layers oxidized [20,21], as expected from the observed high MR ratio. The reason for the absence of TMR effect however, is not clear since the role of the MgO layer on the growth of the GaO*_x_* tunnel-barrier has not been clarified yet. 

In this study, we performed detailed structural studies on the epitaxial MTJ structure to clarify the effects of the MgO underlying layer on the growth of the GaO*_x_* barrier layer as well as their influence upon the TMR effect. 

## 2. Experimental Procedures

**Figure 1:**
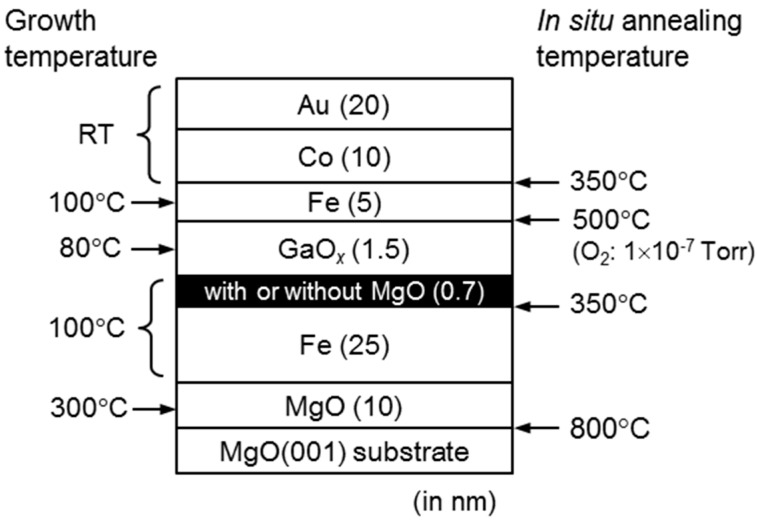
The structure of the magnetic tunnel junction (MTJ) stack designed for this study. Figures at both sides show growth temperatures and in situ annealing conditions.

MTJ films as shown in Figure 1 were grown by molecular beam epitaxy (MBE) in the identical type of growth chamber as mentioned in our previous study [20,21]. 

In growing oxide layers, single-crystal Ga_2_O_3_ and MgO blocks were used as source materials. Prior to the growth, the MgO(001) substrate was heated at 800 °C for surface cleaning. Then, the MgO buffer layer and Fe bottom electrode are respectively grown at 300 and 100 °C, followed by an in situ annealing at 350 °C for 10 min under an ultra-high vacuum (<1 × 10^−9^ Torr) to improve surface morphology of the Fe bottom electrode. After the growth of a 0.7 nm-thick MgO underlying layer (~3 MLs), a GaO*_x_* layer (~1.5 nm) was deposited on the Fe bottom electrode at 80 °C under an O_2_ pressure of 1 × 10^−6^ Torr. Because the surface of the GaO*_x_* layer in the as-grown state is amorphous [21], an in situ annealing for crystallizing the surface region of the GaO*_x_* layer was carried out at temperatures up to 500 °C under an O_2_ pressure of 1 × 10^−7^ Torr. After the annealing, the Fe upper electrode was grown at 100 °C and then annealed again for 10 min at 350 °C under the vacuum to improve the crystalline quality and morphology. Finally, Co-pinned and Au-cap layers were deposited at RT. The Co layer enhances the coercive force of Fe upper electrode so as to realize the antiparallel magnetization alignment (so called pseudo-spin valve structure). For comparison, we also prepared the same structure but without the MgO underlying layer on the Fe bottom electrode. 

Tunnel junctions (3 × 12 μm^2^) for the magneto-transport measurements were fabricated using conventional micro-fabrication techniques [20,21]. Magneto-transport properties of the tunnel junction were measured using a conventional two probe method. The magnetic fields were applied parallel to the major axis of the junction corresponding to the easy axis of the magnetization direction of the FM electrodes.

## 3. Results and Discussions

**Figure 2:**
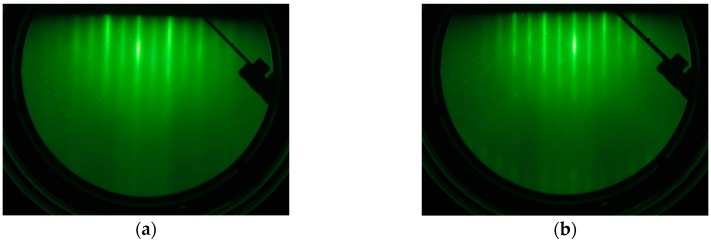
Reflection high-energy electron diffraction (RHEED) images of the GaO*_x_* layer grown on the (**a**) MgO underling layer and (**b**) Fe bottom electrode after an *in-situ* annealing ([110] azimuth of MgO substrate).

Figure 2a,b show the reflection high-energy electron diffraction (RHEED) images of the GaO*_x_* layer for the MTJ samples with or without the MgO underlying layer respectively, after the in situ annealing, with the temperature being raised up to 500 °C under the O_2_ atmosphere. 

**Figure 3:**
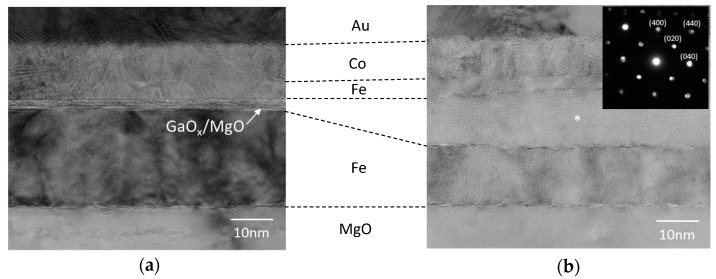
Cross-sectional transmission electron microscope (TEM) images of the MTJ samples (**a**) with (**b**) without the MgO underlying layer ([100] azimuth of the MgO substrate). Broken lines indicate the interfaces among the layers. The inset of Figure 3b shows electron nano-beam diffraction pattern at the point indicated by an asterisk symbol.

The RHEED images of both samples showed similar streaky patterns, indicating the formation of single-crystalline layer with an atomically flat surface in both samples. Also, very similar streaky patterns appeared in the image of the Fe upper electrodes after the annealing in the vacuum. No clear difference from the RHEED observations was observed between both samples. We found however, a remarkable difference in cross-sectional transmission electron microscopy (TEM) images between both samples as given in Figure 3a,b. 

For the sample with MgO underlying layer, a fully epitaxial Fe/GaO*_x_*/(MgO)/Fe structure was recognized as shown in Figure 3a. Total thickness of the GaO*_x_*/MgO layers was estimated to be about 2.2 nm which is close to the designed total thicknesses of the MgO (0.7 nm) and GaO*_x_* (~1.5 nm) layers. In the case without the MgO underlying layer (Figure 3b), on the other hand, a thick (~15 nm) unknown single-crystalline layer appeared between the Fe upper and bottom electrodes. Note that the thickness of the Fe bottom electrode largely decreased due to the formation of the thick unknown layer. These results imply that a part of the Fe bottom electrode was oxidized and intermixed with Ga caused by the in situ annealing at high temperature up to 500 °C under the O_2_ atmosphere. 

Electron nanobeam diffraction (NBD) patterns revealed that the unknown layer (the inset of Figure 3b) could be assigned as a cubic MgAl_2_O_4_-type spinel structure which is identical to that of the GaO*_x_* tunnel-barrier. Crystal orientations of the Fe electrodes and the spinel layer were also determined as upper Fe(001)[110] ǁ spinel (001)[100] ǁ bottom Fe(001)[110] from the NBD analysis. The observed crystal orientations of the Fe electrodes and oxide (spinel) layer are identical to those of epitaxial Fe/γ-GaO*_x_*/(MgO)/Fe MTJ [20,21]. Therefore, it is not surprising that there is no clear difference in the RHEED images between the samples with or without the MgO underlying layer.

**Figure 4:**
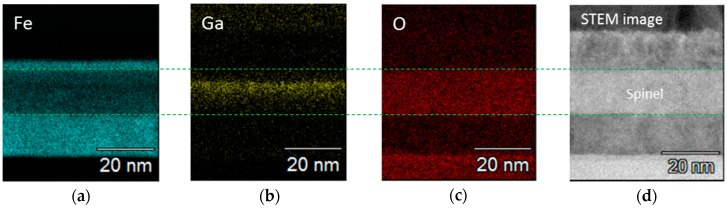
Elemental mappings of (**a**) Fe, (**b**) Ga, and (**c**) O obtained from the MTJ without the MgO underlying layer using an energy-dispersive X-ray spectroscopy (EDX); (**d**) bright-field scanning TEM image of the same area. Broken lines indicate the interfaces between the spinel layer and Fe electrodes.

We performed a composition analysis in the vicinity of the spinel layer by an energy-dispersive X-ray spectroscopy (EDX) as displayed in Figure 4a–c, together with the cross-sectional scanning TEM image observation (Figure 4d) as the EDX analysis. 

The spinel layer clearly consists of Fe, Ga, and O. Large amount of the distributions of Ga and Fe compositions were detected within the layer whereas O composition was also being detected with uniform distribution. This suggests that (Fe, Ga)_2_O_3_ and (Fe, Ga)_3_O_4_ are the possible materials for the observed spinel layer. It should be mentioned here that we have observed in a similar structure as the present sample with the MgO underlying layer, that there are the two distinct layers of GaO*_x_* and MgO without being subjected to losing the thickness of the Fe bottom electrode [20]. The results indicate that a very thin (~3 MLs) MgO underlying layer effectively acts as an oxygen-preventing layer to the Fe bottom electrode during the annealing under the O_2_ atmosphere. 

TMR effect was only observed in sample with MgO underlying layer. The observed MR ratio was 91% at RT, which is close to the reported values in the epitaxial Fe/GaO*_x_*/(MgO)/Fe MTJs [20,21] and several times larger than those of the MTJs with an amorphous GaO*_x_* barrier [22,23,24]. For the sample without the MgO layer however, no TMR effect was observed down to 20 K. We observed a metallic behavior in temperature dependence of the junction resistances, i.e., the junction resistance decreased with decreasing temperature. Moreover, although there is a 15 nm-thick Ga-Fe-O spinel layer between the electrodes, the junction resistances were almost comparable to the parasitic resistance (~10 Ω at RT) which mainly comes from the resistance of the Fe bottom layer. The results imply that the spinel layer has poor electrical characteristics as an insulator.

## 4. Conclusions

We have grown epitaxial Fe/GaO*_x_*/(MgO)/Fe(001) MTJ structure with or without the MgO underlying layer and investigated the effects of the MgO layer on the growth of GaO*_x_* tunnel barrier together with their influence on the TMR effect. It was demonstrated that, when the MgO layer is absent, a thick Ga-Fe-O layer having a spinel-type crystal structure was formed by conducting in situ annealing with the temperature being raised up to 500 °C under the O_2_ atmosphere. As a result, no TMR effect was observed in the sample without the MgO layer. The results indicate that the MgO underlying layer effectively prevents the Fe bottom electrode from oxidation during the annealing process.
